# Predictors of mobility domain of health-related quality of life after rehabilitation in Parkinson’s disease: a pilot study

**DOI:** 10.1186/s40945-018-0051-2

**Published:** 2018-12-27

**Authors:** Thomas Bowman, Elisa Gervasoni, Riccardo Parelli, Johanna Jonsdottir, Maurizio Ferrarin, Davide Cattaneo, Ilaria Carpinella

**Affiliations:** IRCCS Fondazione Don Carlo Gnocchi, Via Capecelatro 66, 20148 Milan, Italy

**Keywords:** Neurological disease, Parkinson, Neurorehabilitation, Prediction, Health-related quality of life, Participation

## Abstract

**Background:**

Parkinson’s disease impacts health-related quality of life (HRQoL), however no studies inquired on predictors of HRQoL changes after rehabilitation. This study assessed the relationship between mobility domain of HRQoL measured by Parkinson’s Disease Questionnaires-39 (PDQ-39) and clinical-demographic characteristics and developed a model predicting changes after rehabilitation.

**Methods:**

Subjects with Parkinson’s disease underwent rehabilitation treatment and completed the following predictors: 10-m walking test (10MWT), Timed Up and Go (TUG), Berg Balance scale (BBS), Activities-specific Balance Confidence scales (ABC), Freezing of Gait (FOGQ) and PDQ-39. Two general linear models were calculated to predict the relationship between HRQoL at baseline and to predict HRQoL changes after rehabilitation.

**Results:**

Forty-two subjects (age 74.9 ± 7.3 years, Hoehn&Yahr 2.8 ± 0.6) completed the baseline evaluation. The first model (multiple R^2^ = 0.59, F = 5.86, *P* < 0.001) showed that ABC (B = − 0.51, CI = − 0.86 to 0.15, R^2^ = 0.41, *P* = 0.005) and FOGQ (B = 2.38, CI = 1.03 to 3.73, R^2^ = 0.07, *P* = 0.001) were statistically significant predictors of mobility aspect of HRQoL at baseline. Thirty seven subjects completed the rehabilitation sessions, data were entered in the second model (multiple R^2^ = 0.40, F = 4.24, *P* < 0.004) showing that gender (B = − 5.12, CI = − 9.86 to − 0.39, R^2^ = 0.23, *P* = 0.034), Hoehn&Yahr (B = 10.93, CI = + 3.27 to + 18.61, R^2^ = 0.22, *P* = 0.006) and PDQ-39 mobility at baseline (B = − 0.38, CI = − 0.63 to − 0.14, R^2^ = 0.55, *P* = 0.002) were statistically significant predictors of changes of the mobility aspect of HRQoL.

**Conclusions:**

Balance confidence and Freezing of Gait are associated with the mobility aspect of HRQoL. Changes in mobility domain of HRQoL (as assessed by PDQ-39) are likely to be greater in males, in people at higher stages of the disease and in people with more severe limitation in mobility domain of HRQoL (as assessed by PDQ-39) before rehabilitation. Results might be different when considering different outcomes or different measures for the same outcome (performance mobility test instead of self-report questionnaires). Further investigations are needed to better understand other components of HRQoL in addition to mobility.

**Trial registration:**

NCT02713971 registered March 8, 2016.

## Background

Parkinson’s Disease (PD) is a common chronic neurodegenerative disease affecting 1% of the population over 60 years of age with incidence and prevalence 1.5 to 2.0 times higher in men than in women [1]. PD affects physical, mental and psychosocial health, impacting quality of life (QoL) [2, 3]. QoL is a multi-dimensional construct defined as the individual self-perceived life function. [3, 4] Most of the existing questionnaire used to describe and assess subjects’ QoL actually measure Health-Related QoL (HRQoL) defined as the aspects of QoL most affected by ill health and self perceived health status. [5] Recent studies found that several health-related factors such as disease severity, disability, gait impairments, complications arising from medication therapy, depressive symptoms, psychosocial well-being and autonomic dysfunction are important contributors to HRQoL in people with PD [6, 7].

Specific tools have been developed to measure HRQoL in people with PD and to assess emotional status, cognitive functions and life’s health before and after rehabilitation [3]. The Parkinson’s Disease Questionnaire (PDQ-39) is a widely used tool to evaluate different domains related to HRQoL in people with PD, including mobility, activities of daily living, emotional well-being, stigma, social support, cognition, communication and pain [8]. These domains of HRQoL have been found related to subjects’ demographic and clinical characteristics such as age, gender, disease severity, disease duration, motor and non-motor PD specific symptoms, and subsequent limitations in mobility and gait [9].

Mobility and gait limitations are major issues for people with PD and these clinical characteristics affect their daily life activities and participation in society. Several studies have been carried out to understand the relationship between mobility, gait limitations and HRQoL with mixed results [10, 11]. Other authors [3] showed that clinical characteristics (dynamic balance and executive function) result as significant predictors of HRQoL but this finding was in contrast by previous studies [6, 7] and the current literature is not exhaustive with respect to this topic. Further, the effect of other clinical characteristics of mobility on HRQoL, such as self-perception of walking and balance abilities, that were not considered in previous studies, need further investigation. Finally we know that studies assessing HRQoL tend to use questionnaires reducing this multi-dimensional construct as a summary index [5] but no studies have investigated the relationship between the mobility components of HRQoL construct (measured by PDQ-39 mobility domain) and the clinical and demographic characteristics.

Since mobility domain of PDQ-39 is a fundamental component of HRQoL construct we hypothesize that knowledge about clinical and demographic characteristics associated to mobility domain of PDQ-39 will be useful for physiotherapist and clinicians to better understand the component of the HRQoL related to mobility and the complexity of the whole construct.

In addition, no previous studies inquired on variables that could predict changes in HRQoL after rehabilitation. Since in the rehabilitation field it has become increasingly important to understand what is associated to improvement in HRQoL, we hypothesize that clinical and demographic characteristics can predict changes in the mobility domain of PDQ-39 measured before and after rehabilitation to predict which subjects will improve in this domain after rehabilitation. For this reason, predictive models could be performed to improve the design of interventions by providing a framework of individual characteristics and parameters prone to change thus improving estimation of patient care needs and improvement in therapeutic plans. Furthermore, predictive models could be used to provide sample size estimates for trials aimed at improving HRQoL in people with PD.

In keeping with our research hypothesis, the aims of this pilot study were to: 1) assess the relationship between HRQoL mobility domain of PDQ-39 at baseline and clinical and demographic characteristics of a sample of people with PD, and 2) develop a model to predict changes in HRQoL mobility domain of PDQ-39 after rehabilitation.

## Methods

### Design

This study combines a cross-sectional design to assess the relationship between HRQoL mobility domain of PDQ-39 at baseline and clinical and demographic characteristics of a sample of people with PD and a longitudinal design (assessments pre and post treatment) to develop a model to predict changes in HRQoL mobility domain of PDQ-39 after rehabilitation.

### Subjects

Forty-two people with PD were consecutively recruited for the study between 2013 and 2015. The eligible population included all PD (inpatient and outpatient) living in the catchment areas requiring rehabilitation. Inclusion criteria were: subjects older than 18 years, Hoehn & Yahr stage between I and IV, ability to walk for 6 m with or without walking aids, ability to maintain standing position for at least 10s but inability to stay on one leg stance more than 10s, having mini-mental state examination (MMSE) > 24 to be able to fulfill patients reported outcome and stable drug therapy (to be monitored throughout the treatment period). People with PD who had deep brain stimulation surgery, mini-mental state examination (MMSE) < 24 and changed drug therapy before the end of the treatment were excluded.People with PD underwent individual training consisting of 20 sessions of 45 min each, 3 times a week. They received balance and gait exercises defined by clinical staff for each patient with or without biofeedback. Exercises were performed in different sensory conditions and/or including a dual-task. Few minutes of muscle stretching, and mobilization exercises were also provided. Each session was performed while subjects were in the “on” medication state, for further information on the study protocol see Carpinella et al [12]

Subjects signed an informed consent form before the beginning of the study. The study was approved by the Ethical Committee of Don Gnocchi Foundation.

### Clinical assessment

Subjects were assessed by a rater before and after the 20 rehabilitation sessions. Each assessment was performed while subjects wore normal shoes and were in the “on” medication state.

The clinical assessment included: 1) The 10-m walking test (10MWT) used to assess gait speed. Subjects walked with or without walking device at a preferred walking speed along a 10-m walkway. The time required to cover the middle 6 m of the walkway was recorded [13]; 2) The Timed Up and Go (TUG) test, used to assess mobility and dynamic balance, by measuring the time taken by the subject to rise from a chair, walk 3 m, turn around, walk back to the chair and sit down [14]; 3) The Berg Balance Scale (BBS), used to measure subject’s balance during tasks involving sitting, standing and positional changes. The scale consists of 14-items that rates function on as scale from 0 (worst) to 4 (best). Maximum total score is 56 [15]; 4) The Activities-specific Balance Confidence (ABC) questionnaire, used to assess balance confidence and balance self-perception. Score range is from 0 to 100, where 100 means high self-perception in balance skills [16]; 5) The Freezing of Gait Questionnaire (FOG-Q), used to assess freezing during walking and its severity. It consists of six items with a score ranging from 0 to 24, where 0 means no freezing [17]; 6) The Unified Parkinson Disease Rating Scale-Motor Section (UPDRS III), used to assess disease severity and disease-specific impairments, through 27 motor items about the clinical spectrum of motor symptoms typical of PD, such as tremor, rigidity, bradykinesia and impairment of axial motor function [6]; 7) The Mini Mental State Examination (MMSE) is used to assess cognitive aspects of mental functions and consists of 11 simple questions or tasks grouped into 7 cognitive domains. The score ranging from 0 to 30 and score of < 24 is the generally an accepted cutoff indicating the presence of cognitive impairment [18] and 8) The Parkinson’s Disease Questionnaire (PDQ-39), used to evaluate HRQoL in people with PD. It consists of 39 items divided in 8 domains, items 1 to 10 for mobility, items 11 to 16 for activities of daily living, items 17 to 22 for emotional well-being, items 23 to 26 for stigma, items 27 to 29 for social support, items 30 to 33 for cognition, items 34 to 36 for communication and items 37 to 39 for pain. Scores for each domain are expressed as a percentage (100% indicating greater dissatisfaction/disruption within a domain). The total score is computed by summing the 8 domain scores divided by the total number of domains. In the present study, the mobility domain of the PDQ-39 was used to describe the mobility aspect of HRQoL [8, 19].

### Statistical analysis

Parametric descriptive statistics were used to describe demographic and clinical characteristics of the sample and to detect the presence of outliers. Data distribution was checked for normality and data with a skewness score greater than 1 or − 1 were transformed, BBS cubed scores and TUG logarithm scores were calculated. Paired T test was used to compare general improvement of PDQ-39 mobility domain before and after rehabilitation.

Aim 1) To assess the relationship between HRQoL mobility domain of PDQ-39 at baseline and clinical and demographic characteristics the Hosmer and Lemeshow [20] two-step approach has been used to decrease redundancy and to reduce number of predictors due to small sample size. In the first step univariate analyses (Pearson correlation coefficient and independent sample T test) between mobility domain of PDQ-39 at baseline and clinical and demographic characteristics were calculated. In the second step (multivariate analysis) only variables associated at univariate analyses (*P* < 0.2 with PDQ-39 mobility domain at baseline) were entered in a general linear model as independent variables while PDQ-39 mobility domain score at baseline was used as dependent variable.

Aim 2) The same two-step statistical approach [20] of the first aim has been used to develop a model to predict changes in HRQoL mobility domain of PDQ-39 after rehabilitation. In the first step univariate analysis (Pearson correlation coefficient and independent sample T test) was calculated between PDQ-39 mobility domain change score (post rehabilitation score - baseline score) and demographic and clinical variables (including PDQ-39 mobility domain at baseline) to calculate associations between HRQoL changes and clinical predictors. In the second step (multivariate analysis) clinical predictors associated at univariate analysis) were entered in the second general linear model as independent predictors while PDQ-39 mobility domain change score was used as dependent variable. For both models we checked for collinearity (variance inflation factor < 6), distribution of residuals and influential points and the level of statistical significance was set at *P* < 0.05. Statistical analyses were performed using STATISTICA 9.0.

## Results

Demographic and clinical characteristics of the recruited sample (28 males and 14 females) are shown in Table 1.

**Table 1:** Demographic and clinical characteristics of the sample

Measure	Mean (SD)	Max	Min
AGE (years)	74.9 (7.3)	89.8	57.56
TIME FROM ONSET (years)	9.2(5.0)	21.3	0.44
PDQ-39	31.7(14.1)	63.8	7.1
PDQ-39 mobility domain	48.3(25.2)	95	2.5
H&Y	2.8(0.6)	4	2
10MWT (s)	13.3(7.4)	50.3	6.94
TUG (s)	19.5(12.9)	72	7
BBS	44.2(9.7)	56	17
ABC	52.4(21.6)	97.5	13.13
FOGQ	12.6(4.4)	21	2
UPDRS part 3	19.8(7.3)	37	4
MMSE	27.6(1.9)	30	22.4

Data are represented as means, standard deviations (SD), max and min values

*PDQ-39* Parkinson’s Disease Questionnaire, *PDQ-39 mobility domain* Parkinson’s Disease Questionnaire - mobility domain, *H&Y* Hoehn and Yahr, *10MWT* 10-m walking test, *TUG* Timed up and go, *BBS* Berg Balance Scale, *ABC* Activities Balance Confidence, *FOGQ* Freezing of gait questionnaire, *UPDRS part 3* Unified Parkinson Disease Rating Scale-Motor Section part 3, *MMSE* Mini Mental State Examination

### Aim1-assessment of the relationship between HRQoL mobility domain of PDQ-39 at baseline and clinical and demographic characteristics

Table 2 shows correlations and *p*-values between demographic and clinical characteristics and PDQ-39 mobility domain at baseline.

**Table 2:** Correlations (Pearson) between demographic and clinical variables and PDQ-39 mobility domain at baseline

	PDQ-39 mobility domain (baseline)	*P*-value
GENDER	0.37	0.019*
AGE (years)	0.12	0.452
TIME FROM ONSET (years)	0.15	0.357
H&Y	0.38	0.015*
10MWT(s)	0.46	0.003*
TUG(s)	0.47	0.002*
BBS	−0.40	0.009*
ABC	− 0.63	< 0.001*
FOGQ	0.53	< 0.001*
UPDRS part 3	0.28	0.073*
MMSE	−0.06	0.729

*PDQ39 mobility domain* Parkinson’s Disease Questionnaire-mobility domain, *H&Y* Hoehn and Yahr, *10MWT* 10-m walking test, *TUG* Timed up and go, *BBS* Berg Balance Scale, *ABC* Activities Balance Confidence, *FOGQ* Freezing of gait questionnaire, *UPDRS part 3* Unified Parkinson Disease Rating Scale-Motor Section part 3, *MMSE* Mini Mental State Examination. * *P*-value < 0.2

Males had statistically significant lower scores on the PDQ-39 mobility domain compared to female (Male: 41.9 ± 24.7, Female 61.1 ± 21.7, *P* = 0.02). Correlations between PDQ-39 mobility domain at baseline and gender; H&Y, 10MWT, TUG, BBS, ABC, UPDRS part 3 and FOGQ (*P* < 0.2) were entered in the multivariate model (Table 3) investigating effect of baseline values on mobility aspect of HRQoL. No statistically significant correlations were found between PDQ-39 mobility domain at baseline and age, time from onset and MMSE (Table 2) therefore these variables were not entered in the model. The general linear model fitted the data (multiple R^2^ = 0.59, adjusted R^2^ = 0.49, F = 5.86, *P* < 0.0001) and the ABC (B = − 0.51, CI = − 0.86 to 0.15, R^2^ = 0.41, *P* = 0.005) and the FOGQ (B = 2.38, CI = 1.03 to 3.73, R^2^ = 0.07, *P* = 0.001) were significantly associated with the PDQ-39 mobility domain at baseline.

**Table 3:** Model analysis between demographic and clinical variables and PDQ-39 mobility domain at baseline

DEPENDENT VARIABLE	multiple R^2^	adjusted R^2^	F	*P* value	INDEPENDENT VARIABLES	Β	CI (−95 to + 95%)	R^2^	*P* value
PDQ-39 mobility domain (baseline)	0.59	0.49	5.86	< 0.0001*	Gender	−2.05	−9.34 to + 5.24	0.30	0.571
					H&Y	0.01	−15.55 to + 15.58	0.58	0.998
					TUG	36.16	−29.78 to + 102.1	0.84	0.272
					10MWT	−9.50	−86.31 to + 67.30	0.81	0.802
					BBS	0.00004	−0.002 to + 0.003	0.68	0.758
					ABC	−0.51	− 0.86 to + 0.15	0.41	0.005*
					FOGQ	2.38	+ 1.03 to +3.73	0.07	0.001*
					UPDRS part 3	−0.26	−1.45 to + 0.91	0.52	0.645

*PDQ-39 mobility domain* Parkinson’s Disease Questionnaire mobility domain, *H&Y* Hoehn and Yahr, *TUG* Timed up and go, *10MWT* 10-m walking test, *BBS* Berg Balance Scale, *ABC* Activities Balance Confidence, *FOGQ* Freezing of gait questionnaire, *UPDRS part 3* Unified Parkinson Disease Rating Scale-Motor Section part 3. * *P*-value < 0.05

### Aim2-development of a model to predict changes in HRQoL mobility domain of PDQ-39 after rehabilitation

Table 4 shows correlations and *p*-values between demographic and clinical characteristics and PDQ-39 mobility domain change score.

**Table 4:** Correlations (Pearson) between demographic and clinical variables and PDQ-39 mobility domain change score

	PDQ-39 mobility domain (change score)	*P*-value
GENDER	0.21	0.20*
AGE (years)	0.14	0.41
TIME FROM ONSET (years)	−0.15	0.39
H&Y	0.22	0.20*
10MWT(s)	0.09	0.58
TUG(s)	−0.04	0.81
BBS	−0.06	0.70
ABC	0.09	0.58
FOGQ	−0.27	0.10*
UPDRS part 3	0.01	0.93
MMSE	0.23	0.17*
PDQ39 mobility domain (baseline)	−0.37	0.02*

*PDQ39 mobility domain* Parkinson’s Disease Questionnaire-mobility domain, *H&Y* Hoehn and Yahr, *10MWT* 10-m walking test, *TUG* Timed up and go, *BBS* Berg Balance Scale, *ABC* Activities Balance Confidence, *FOGQ* Freezing of gait questionnaire, *UPDRS part 3* Unified Parkinson Disease Rating Scale-Motor Section part 3, *MMSE* Mini Mental State Examination. * *P*-value < 0.2

No statistically significant correlations were found between PDQ-39 mobility domain change score and age, time from onset, 10MWT, TUG, BBS, ABC and UPDRS part 3 (Table 4). Statistically significant correlations (*P* < 0.2) were instead found between PDQ-39 mobility change score and gender, H&Y, FOGQ, MMSE and PDQ-39 mobility domain at baseline and these variables were entered in the multivariate model.

Change score on the PDQ-39 mobility domain was − 6.2 (±14.6) points suggesting an overall improvement in HRQoL after rehabilitation (Paired T Test, *T* = 2.56, *P* = 0.015).

Five subjects were missing at post assessment resulting in data of thirty-seven subjects being included in the second multivariate model (Table 5) predicting rehabilitation outcome of HRQoL in people with PD.

**Table 5:** Model analysis between demographic and clinical variables and PDQ-39 mobility domain change score

DEPENDENT VARIABLE	Multiple R^2^	Adjusted R^2^	F	P value	INDEPENDENT VARIABLES	B	CL (−95 to + 95%)	R^2^	P value
PDQ-39 mobility domain (change score)	0.40	0.31	4.24	0.004*	Gender	−5.12	−9.86 to −0.39	0.23	0.034*
					H&Y	10.93	+3.27 to + 18.61	0.22	0.006*
					MMSE	1.21	−1.17 to + 3.60	0.018	0.307
					FOGQ	−0.13	− 1.28 to + 1.03	0.36	0.824
					PDQ-39 mobility domain (baseline)	−0.38	−0.63 to − 0.14	0.55	0.002*

*PDQ-39 mobility domain* Parkinson’s Disease Questionnaire mobility domain, *H&Y* Hoehn and Yahr, *MMSE* Mini Mental State Examination, *FOGQ* Freezing of gait questionnaire. * *P*-value < 0.05

The general linear model fitted the data (multiple R^2^ = 0.40, adjusted R^2^ = 0.31 F = 4.24, *P* < 0.004) and Gender (B = − 5.12, CI = − 9.86 to − 0.39, R^2^ = 0.23, *P* = 0.034), H&Y (B = 10.93, CI = + 3.27 to + 18.61, R^2^ = 0.22, *P* = 0.006) e PDQ-39 mobility domain at baseline (B = − 0.38, CI = − 0.63 to − 0.14, R^2^ = 0.55, *P* = 0.002) were significant predictors of a change on the PDQ-39 mobility domain after rehabilitation.

## Discussion

The aim of this study was to develop two models with baseline demographic and clinical variables predicting, respectively, to mobility-relate HRQoL and its change after rehabilitation in people with PD. The main results indicate that mobility aspect of HRQoL is mostly associated with subject’s perception of gait and balance disorders (measured by FOG-Q and ABC questionnaires respectively). Conversely, improvement in mobility related HRQoL following rehabilitation was predicted by a worse PDQ-39 mobility domain at baseline, higher disease severity, and being male suggesting that even subjects in their later stage of the disease can improve mobility related QoL. This information is useful to define criteria to include people with PD in rehabilitation program having mobility related HRQoL as the main outcome, to set rehabilitation goals and to identify causes of failure to recover.

### Aim1-assessment of the relationship between HRQoL mobility domain of PDQ-39 at baseline and clinical and demographic characteristics

Univariate correlation analysis showed moderate correlation between mobility related HRQoL and gait and balance disorders. This was true both for self-administered tests inquiring on subject’s perception of their balance confidence (ABC) and freezing of gait (FOG-Q), and rater-administered tests assessing static and dynamic balance (BBS and TUG) and walking skills (10MWT). Conversely, mild correlations were found between mobility aspect of HRQoL and overall disability (H&Y and UPDRS III), cognitive function (MMSE) and demographic characteristics (age and time from onset), suggesting that limitations in walking and balance are more specifically associated to mobility related HRQoL [3]. These results are in line with previous studies [9, 21–25] investigating parameters affecting HRQoL in people with PD. Some authors [9] found that postural instability and gait disorders predicted overall HRQoL, others [23] pointed out that mobility disorders, mostly start hesitation, freezing, festination and difficulty in turning, are related to HRQoL.

When predictors were entered in the first general linear model balance self-perception (ABC) and freezing of gait (FOG-Q) were better predictors of mobility aspect of HRQoL than rater-administered tests, even after controlling for all the other variables included in the model. Along those lines, previous studies [26–29] found that balance self-perception is associated with fear of falling in people with PD and is one of the main predictors of HRQoL. The relationship between balance self-perception, participation restrictions and HRQoL is also supported by studies suggesting that people with PD with low balance self-perception as measured by the ABC are more likely to use an assistive device to walk [27, 30] to improve the sense of safety increasing their mobility, independence and, consequently, HRQoL.

Besides low level of balance confidence, also freezing of gait was associated to lower mobility related to HRQoL. This result is confirmed by previous studies showing the impact of freezing on QoL in PD [31, 32]. Some authors [31, 33], showed that HRQoL decreases proportionally with the severity of FOGQ scores and found that freezing has an independent, direct and significant impact on HRQoL in people with PD even controlling for gait and mobility disorders. This can be due to the nature of freezing, consisting in an episodic event that causes a sudden and unpredictable inability to maintain walking [32]. Often, people with PD are not prepared for this event that can lead to perceived loss of control on their own body, compromising mobility and leading to loss of independence and increased risk of falling [31, 34, 35].

Moreover, freezing of gait can have social consequences because frequent episodes in crowded situations, during social events or activities of everyday life become a source of stress, embarrassment and frustration with consequences on emotional well-being [31, 36, 37]. Thus, it is possible that loss of control and motor difficulties caused by freezing of gait, combined with psychological distress, are reflected in a worse mobility related QoL. These findings underline the importance of efforts to alleviate freezing of gait and its related consequences, such as the negative impact on mobility aspect of HRQoL in people with PD.

### Aim2-development of a model to predict changes in HRQoL mobility domain of PDQ-39 after rehabilitation

Univariate correlation analysis showed that changes in mobility domain of HRQoL is mostly correlated to HRQoL mobility domain at baseline evaluation. Also, the degree of disability (H&Y), cognitive function (MMSE) and demographic characteristics (gender) were correlated with changes in mobility aspect of HRQoL.

The results of the second general linear model taking in to account univariate correlations showed that the PDQ-39 mobility domain at baseline, severity of disease (H&Y) and gender were significant predictors for changes in HRQoL.

PDQ-39 mobility domain at baseline was found to be the best predictor. In particular those PD patients having worse HRQoL mobility domain at baseline tended to improve more in HRQoL mobility after treatment. This trend was confirmed by studies considering different populations of subjects. For example, Asiri et al. [38] found that the most impaired post-stroke subjects showed larger degree of improvement in gait speed after home-based training in subjects with lower HRQoL related to mobility at baseline. Similarly, Altenburg et al. [39] found larger improvement after cardio-pulmonary rehabilitation in patients affected by chronic obstructive pulmonary disease with low initial exercise capacity. It is possible that PD subjects that were less affected had a floor effect on the PDQ-39 (12% of the sample) masking possible improvements in HRQoL. [40] On the other hand, worse baseline values might indicate a bigger potential for improvement. We can also speculate that people with PD with low HRQoL related to mobility at baseline have entered a downward spiral of avoidance in engaging activities of daily living, thus increasing participation restriction, deconditioning and demotivation. In this context rehabilitation may have increased ability in participating in social events and motivation, maybe decreasing depression. Unfortunately, we did not take in account these psychological and non-motor symptoms that are considered as predictors of HRQoL outcome as reported in recent studies [7, 41–43].

Disease severity was found to be the second best statistically significant predictor for rehabilitation outcome in HRQoL. PD subjects with moderate disease severity (H&Y between 2 and 3), involving axial motor symptoms with balance and gait deficits, seemed to improve more their mobility aspect of HRQoL after rehabilitation. Our results are in line with a systematic review [7] suggesting that disease severity and motor features including gait impairments were the major predictors of poor HRQoL in people with PD in combination with non-motor characteristics as depression and treatment-induced complications. Moreover, contemporary literature [44] pointed out that factors as disease severity influences HRQoL but a better management strategy can slow down or lower their negative effects. A recent study by Rafferty et al., [45] demonstrated that long-term HRQoL benefit following rehabilitation was greater in people having moderate to advanced PD severity compared with those with mild PD severity. It is possible that rehabilitation of the more impaired subjects leads to larger improvement in mobility and consequently in perception of mobility, thus increasing their confidence in performing activities considered too difficult before rehabilitation leading to reduced disability and improved HRQoL.

Gender was the third significant predictor for rehabilitation outcome in mobility aspect of HRQoL. Males had higher PDQ-39 mobility domain change scores following rehabilitation with a mean improvement of − 11.5 points, compared to − 0.4 points for female. As previously demonstrated, people with PD showed gender-related differences in disease experience and HRQoL perception factors that can have important clinical implications [46]. For example, being female has a negative impact on drug and surgical outcome in PD treatments [47–49], with females also showing poorer short and long-term motor outcome after subthalamic stimulation [47, 50]. Despite our results, contemporary literature stated that the effects of demographic characteristics (gender, age, level of education) on HRQoL in PD subjects are controversial [44] and considering our small sample size we should be careful to generalize our results to the whole PD population. Although gender has been shown to influence brain anatomy, function, hormonal modulation, gene expression and levodopa bioavailability [49, 51, 52], further studies are needed to better understand role of gender in rehabilitation outcome.

Keeping into account our results on demographic and clinical (motor) factors and the recent growing evidence demonstrating the impact of non-motor characteristics on lives of people with PD [53] an efficient strategy to maintain and improve HRQoL in people with PD should consist of a holistic, multidisciplinary, personalized and patient-centered approach with timely administration of palliative care and effectual involvement of caregivers and family members [44].

## Limitation and conclusions

First and main limitation of this study is the sample size that was too small and, therefore, the results could not be generalized to all PD subjects and reduced the power of the study. Further studies with larger sample size are needed to get firm conclusion on predictors of HRQoL improvements after rehabilitation. Second, analysis of follow-up data should be included to understand long term predictors of changes in QoL related to mobility. Third, assessments were completed only during on medication state. Evaluation in off-medication state may give more information about the relationship between motor symptoms and mobility aspect of QoL. Fourth, we did not take in account psychological and non-motor symptoms and a small percentage of people with PD (12%) showed floor effects on the PDQ-39, leading to bias in data analysis.

Even with these limitations our study shows that balance confidence and freezing of gait are associated with the mobility aspect of QoL. Changes in mobility (as assessed by PDQ-39) are likely to be greater in males, in people at higher stages of the disease and in people with more severe mobility limitation (as assessed by PDQ-39) before rehabilitation. Results might be different when considering different outcomes or different measures for the same outcome (performance mobility test instead of self-report questionnaires).

Considering that HRQoL is a multi-dimensional construct, further research with larger sample size will be needed to find other predictors of the HRQoL domains in addition to mobility domain.

## Abbreviations

10MWT: 10-m walking test
ABC: Activities Balance Confidence
BBS: Berg Balance Scale
FOGQ: Freezing of gait questionnaire
H&Y: Hoehn and Yahr
HRQoL: Health Related Quality of like
MMSE: Mini Mental State Examination
PDQ39: Parkinson’s Disease Questionnaire
QoL: Quality of life
TUG: Timed up and go
UPDRS part 3: Unified Parkinson Disease Rating Scale-Motor Section part 3
